# Immunophenotype and Response to Immunotherapy of *RET*-Rearranged Lung Cancers

**DOI:** 10.1200/PO.18.00386

**Published:** 2019-05-16

**Authors:** Michael Offin, Robin Guo, Stephanie L. Wu, Joshua Sabari, Josiah D. Land, Ai Ni, Joseph Montecalvo, Darragh F. Halpenny, Larry W. Buie, Terry Pak, Dazhi Liu, Gregory J. Riely, Matthew D. Hellmann, Ryma Benayed, Maria Arcila, Mark G. Kris, Charles M. Rudin, Bob T. Li, Marc Ladanyi, Natasha Rekhtman, Alexander Drilon

**Affiliations:** ^1^ Memorial Sloan Kettering Cancer Center, New York, NY; ^2^ Weill Cornell Medical College, New York, NY

## INTRODUCTION

*RET* rearrangements are identified in 1% to 2% of non–small-cell lung cancers (NSCLCs).^1,2^ In patients with advanced, *RET*-rearranged lung cancers, systemic therapy can be highly active. We demonstrated previously that pemetrexed-based chemotherapy can achieve an objective response rate of 45% and a median progression-free survival (PFS) of 19 months.^3^ Furthermore, the activity of targeted therapy has improved dramatically with the introduction of selective RET inhibitors to the clinic. In early-phase testing, objective response rates with LOXO-292^4^ and BLU-667^5^ are 68% (26 of 38) and 50% (seven of 14), respectively. These outcomes exceed the modest activity observed previously with multikinase inhibitors such as cabozantinib^6^ and vandetanib.^7^

In contrast, the activity of immunotherapy in *RET*-rearranged lung cancers has not been well characterized. This represents a clear unmet need, given that all prior regulatory approvals of immune checkpoint inhibitors, either alone or in combination with chemotherapy, and in stage III or IV disease, have technically included patients with *RET*-rearranged lung cancers.^8,9^ Furthermore, although increasing levels of programmed death-ligand 1 (PD-L1) expression and high tumor mutational burden (TMB) have been associated with benefit from immune checkpoint blockade,^10^ the immunophenotype of *RET*-rearranged lung cancers and the role of PD-L1 and TMB status in relation to benefit with immunotherapy remain poorly described. We set out to characterize these factors.

## METHODS

In this retrospective study, patients from Memorial Sloan Kettering Cancer Center with *RET*-rearranged lung cancers diagnosed between 2009 and 2017 were identified under an institutional review board–approved waiver. Clinical characteristics including age, sex, and smoking history were collected. Patients who received immunotherapy, defined as a monoclonal antibody against programmed cell death protein 1 (PD-1) or PD-L1, were included in an analysis of treatment history.

*RET* rearrangements were identified using targeted next-generation sequencing of DNA (Memorial Sloan Kettering–Integrated Mutation Profiling of Actionable Cancer Targets [MSK-IMPACT] or Foundation One) or RNA (anchored multiplex polymerase chain reaction [PCR]; Memorial Sloan Kettering Solid Fusion Panel) in more contemporary samples, and fluorescence in situ hybridization (10q11 and 6q22 break apart probe, Metasystems, Altussheim, Germany) or reverse-transcriptase PCR in older samples.^11,12^ PD-L1 immunohistochemistry was evaluated using the E1L3N antibody (Cell Signaling Technology, Danvers, MA), our institutional standard, which has been validated against a 22C3 kit performed in a commercial laboratory with comparable results.^13^ For uniformity, TMB (reported as the number of nonsynonymous mutations per megabase) was analyzed only for samples sequenced using MSK-IMPACT.^14,15^ The median TMB of *RET*-rearranged was compared with that of *RET* wild-type NSCLCs (Mann-Whitney *U* test).

In patients with both baseline and serial on-treatment imaging, the best objective response to therapy (Response Evaluation Criteria in Solid Tumors [RECIST] v1.1) was determined by a study radiologist. Time to treatment discontinuation (TTD) was defined as the time from therapy initiation to the last dose.^16^ PFS was defined as the time from therapy initiation to radiologic progression or death. Overall survival (OS) was defined as the time from diagnosis of metastatic disease to death. For TTD, PFS, and OS analyses, Kaplan-Meier curves were compared using the Mantel-Cox log-rank test. Hazard ratios were calculated using the Mantel-Haenszel method.

## RESULTS

Seventy-four patients with *RET*-rearranged lung cancers were identified. Clinicopathologic features are summarized in Table 1. The median age was 58 years, 55% were female, and 69% were never-smokers. *RET* rearrangement was identified as follows: DNA-based next-generation sequencing (80% [n = 59]), RNA-based anchored multiplex PCR (1% [n = 1]), fluorescence in situ hybridization (15% [n = 11]), and reverse-transcriptase PCR (4% [n = 3]). Consistent with previous reports, the most common *RET* fusion partner was *KIF5B* (66% [n = 43 of 65]), followed by *CCDC6* (18% [n = 12 of 65]) when known.

**TABLE 1.**
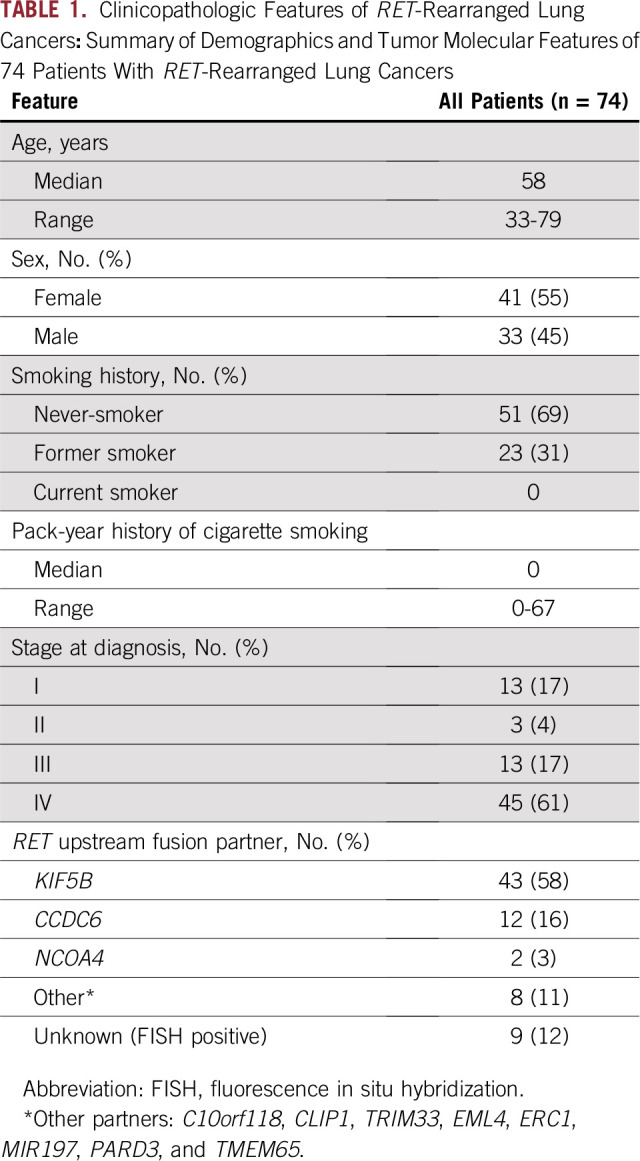
Clinicopathologic Features of *RET*-Rearranged Lung Cancers**:** Summary of Demographics and Tumor Molecular Features of 74 Patients With *RET*-Rearranged Lung Cancers

**FIG 1.**
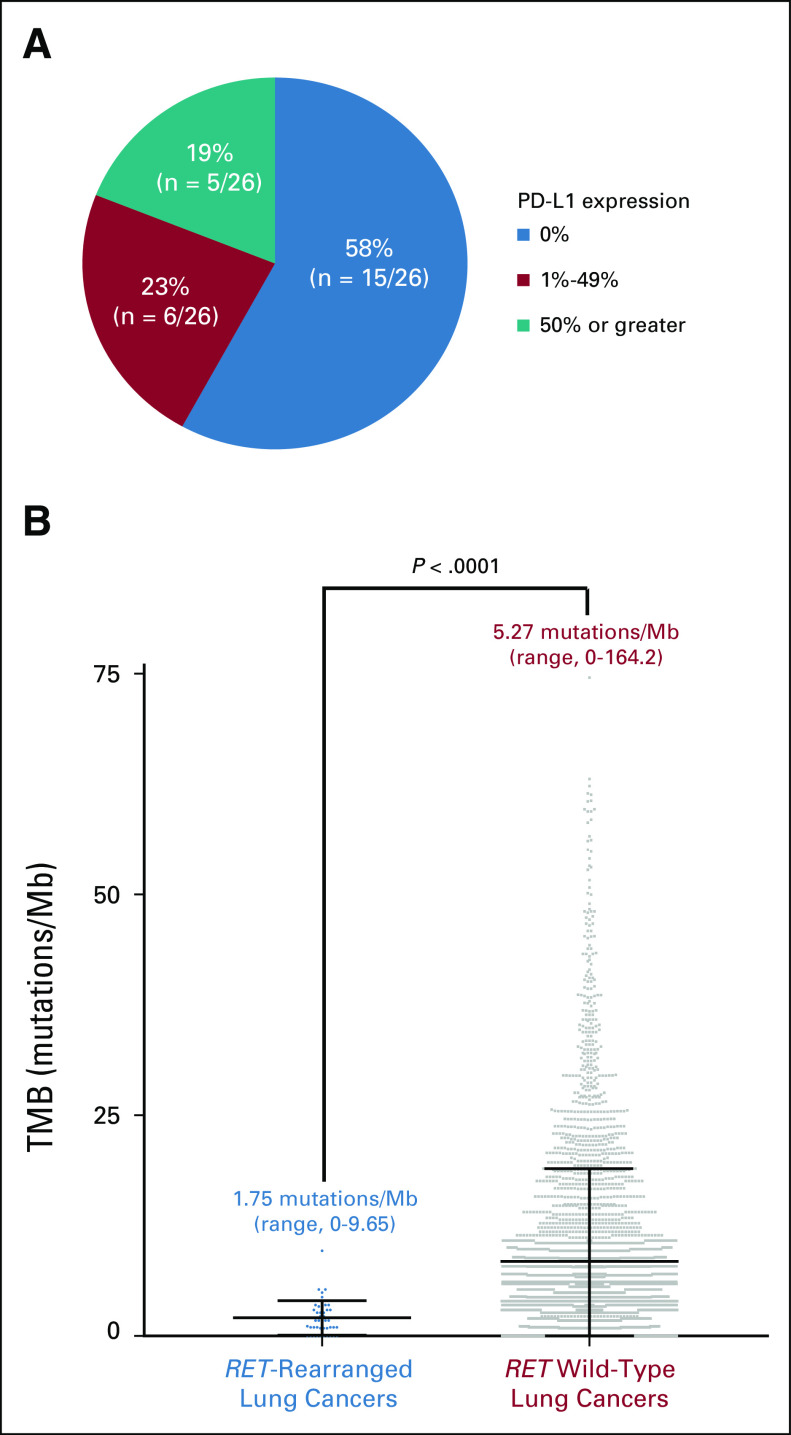
Immunophenotype of *RET*-rearranged lung cancers. (A) The programmed death-ligand 1 (PD-L1) expression (E1L3N, Cell Signaling) of 26 *RET*-rearranged lung cancers with sufficient tissue for testing is shown. The majority (81%) of tumors had either no PD-L1 expression (0%) or low levels of PD-L1 expression (1% to 49%). (B) The tumor mutational burden (TMB) of 44 *RET*-rearranged lung cancers is displayed (left) relative to the TMB of 3,631 *RET* wild-type lung cancers (right). Above each plot, the median TMB and TMB range are indicated. The median TMB of *RET*-rearranged lung cancers was significantly lower than the median TMB of *RET* wild-type lung cancers (Mann-Whitney; *P* < .0001). For ease of representation, three outlier *RET* wild-type lung cancer samples with TMB greater than 75 mutations/Mb that were included in the statistical analysis were excluded in this plot.

In patients with sufficient tissue for PD-L1 testing, tumor PD-L1 expression was 0%, 1% to 49%, and 50% or greater in 58% (n = 15 of 26), 23% (n = six of 26), and 19% (n = five of 26) of cases, respectively (Fig 1A). PD-L1 expression was absent or below 50% in the majority of tumors (81% [n = 21 of 26]). No major differences in PD-L1 expression were observed when stratified by upstream fusion partner (Appendix Table A1). In 44 patients with sufficient tissue for TMB analysis, the median TMB was 1.75 mutations/Mb (range, 0 to 9.65 mutations/Mb), significantly lower (*P* < .0001) than the median TMB of 5.27 mutations/Mb (range, 0 to 164.20 mutations/Mb) in 3,631 patients with *RET* wild-type NSCLCs (Fig 1B).

The clinical outcomes of immunotherapy in patients with advanced *RET*-rearranged lung cancers are summarized in Table 2. Patients received pembrolizumab (n = 6), nivolumab (n = 6), atezolizumab (n = 2), durvalumab (n = 1), or ipilimumab plus nivolumab (n = 1). The median line of therapy at which immunotherapy was administered was 2 (range, 1 to 7). In cases with sufficient tissue for testing, PD-L1 expression ranged from 0% to 50% and TMB ranged from 1.76 to 5.27 mutations/Mb.

**TABLE 2.**
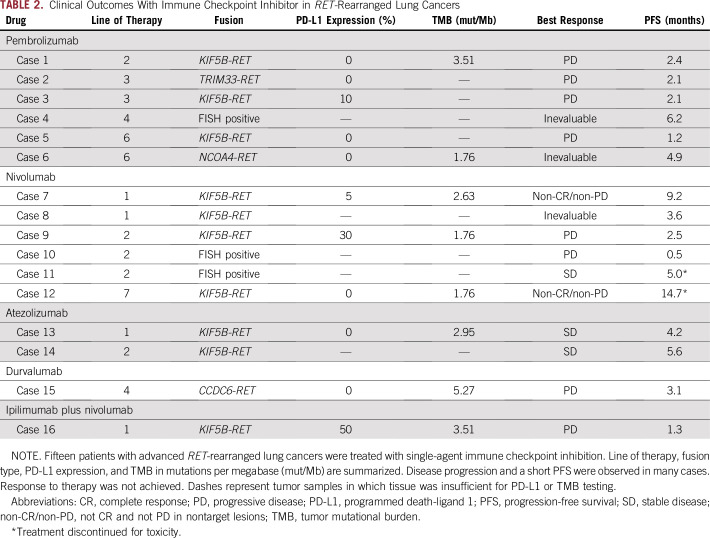
Clinical Outcomes With Immune Checkpoint Inhibitor in *RET-*Rearranged Lung Cancers

**FIG 2.**
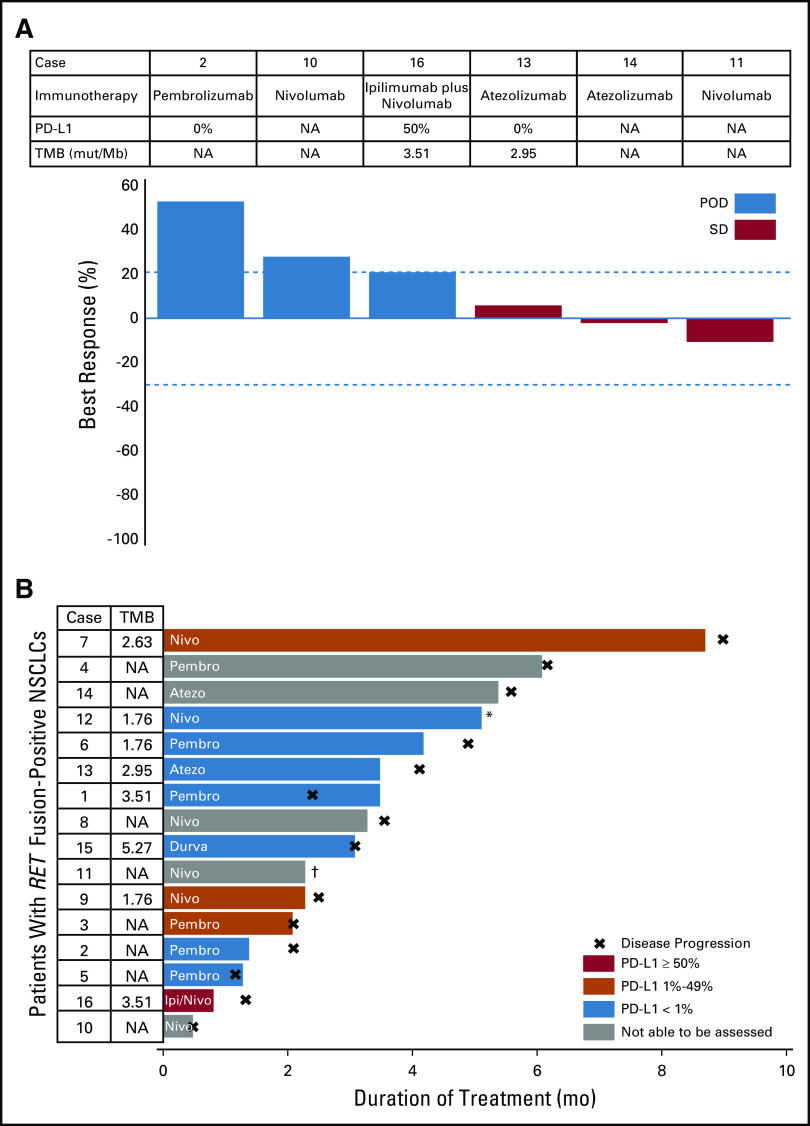
Response to immunotherapy and treatment duration. (A) A waterfall plot of best objective response to single-agent immune checkpoint inhibition is shown. Only patients with measurable disease (n = 6) were included in this analysis. No complete or partial responses were observed. (B) A swimmer’s plot of time to treatment discontinuation is shown for all patients who received immunotherapy (n = 16). None of the patients who received immunotherapy remain on treatment. For both figures, the agent administered and the immunophenotype (programmed death-ligand 1 [PD-L1]) and tumor mutational burden [TMB], when known) are indicated for each patient. (*) Immune checkpoint inhibitor stopped for toxicity; continued stable disease for a PFS of 14.7 months. (†) Immune checkpoint inhibitor stopped for toxicity; continued stable disease for PFS of 5 months, but died as a result of pneumonitis. mut, mutations; NA, not available; NSCLC, non–small-cell lung cancer; POD, progression of disease.

A total of 13 patients with *RET*-rearranged lung cancers were assessed for clinical and/or radiologic response. Response to immunotherapy was not observed. Progression of disease was observed in 62% of cases (n = eight of 13; Table 2); disease progression involved new or worsening brain metastases in three patients (cases 4, 6, and 15). Stable disease was achieved in 23% (three of 13) and non-CR/non-PD (not complete reponse and not progressive disease in nontarget lesions) in 15% (two of 13). A waterfall plot of best objective response in the six patients with measurable disease is presented in Fig 2A.

The median PFS in all patients was 3.4 months (95% CI, 2.1 to 5.6 months). No association was seen between PD-L1 or TMB status and PFS. In patients with the highest levels of PD-L1 expression (50% and 30%), PFS was short (1.3 months and 2.5 months, respectively). Similarly, in patients with the highest TMB (5.27 and 3.51 mutations/Mb), PFS was short (3.1 and 1.3 months, respectively).

**Fig A1.**
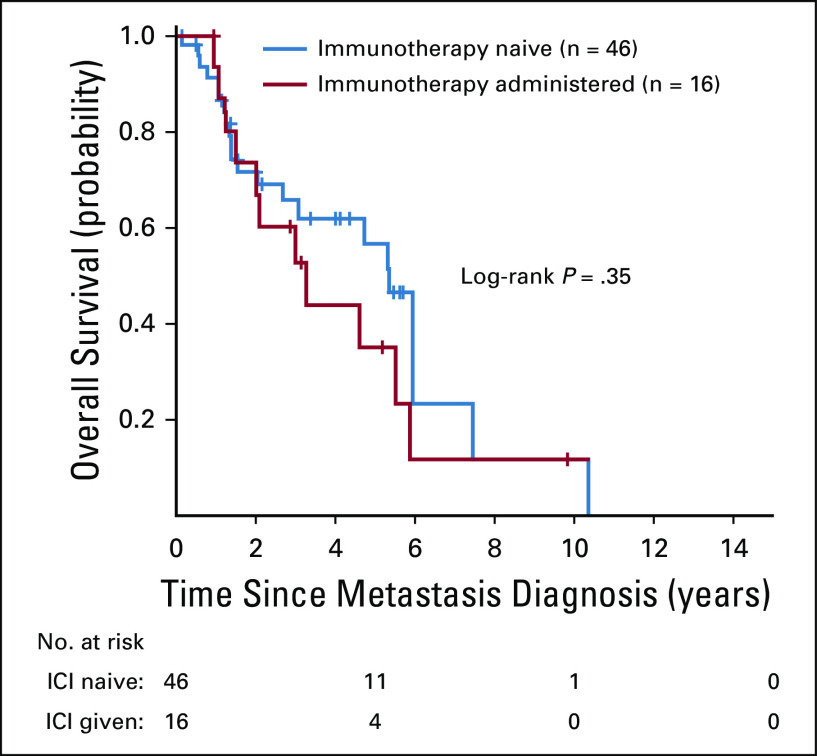
Overall survival of *RET*-rearranged lung cancers. Overall survival from the diagnosis of metastatic disease was compared between patients who received an immune checkpoint inhibitor (n = 16) and those who did not (n = 46). There was no difference in overall survival between these groups (hazard ratio, 1.4 [95% CI, 0.7 to 2.9]; log-rank *P* = .35). ICI, immune checkpoint inhibitor.

A swimmer’s plot of TTD is presented in Fig 2B. The median TTD in all patients was 3.2 months (range, 0.5 to 14.7 months). Treatment was discontinued for toxicity in two of 16 patients (fatigue [case 11] and pneumonitis [case 8]). When patients with advanced *RET*-rearranged lung cancers who received immunotherapy (n = 16) were compared with those who did not receive immunotherapy (n = 46), there was no difference in OS (hazard ratio, 1.4 [95% CI, 0.7 to 2.9]; log-rank *P* = .35; Appendix Fig A1).

## DISCUSSION

In this series, we demonstrate that the immunophenotype of *RET*-rearranged lung cancers is characterized by low levels of PD-L1 expression and low TMB in the majority of patients. This raises the possibility that these tumors are biologically cold (ie, less likely to be highly responsive to immunotherapy relative to other cancers). Consistent with this hypothesis, overall outcomes with single- and dual-agent immunotherapy were poor. No responses were observed, and the best objective response to therapy in most patients was progressive disease. Furthermore, median PFS was short.

These findings are consistent with a growing body of evidence uncovering poor outcomes with immune checkpoint inhibition in select oncogene-addicted lung cancers. In *EGFR*-mutant and *ALK*-rearranged lung cancers, early data on the decreased activity (compared with unselected cancers) of immunotherapy resulted in the exclusion of patients with these tumors in registration-enabling studies. Furthermore, we showed previously that *MET* exon 14–altered NSCLCs had low TMB and poor outcomes with immunotherapy.^17^ Finally, an ongoing global registry (Immunotarget) and independent series have shown similarly low response rates and short median PFS with single-agent immune checkpoint inhibition in lung cancers with oncogenic drivers.^18^ Immunotarget had 16 patients in the *RET*-rearranged cohort and reported a response rate of 6% and median PFS of 2.1 months, comparable to the findings in our study.

The clinical implications of these observations relate to the sequence with which immunotherapy is used and the type of immunotherapy strategy selected. Regarding the former, it is becoming increasingly clear that specific, targeted therapies (selective RET inhibitors)^2,4,19^ and chemotherapy agents (pemetrexed-containing regimens)^3^ can achieve superior outcomes compared with immunotherapy in this series. Thus, it is reasonable to consider the use of checkpoint inhibition only after select targeted therapies and platinum doublet-containing chemotherapy have been administered.

Note that high PD-L1 expression (50% or more) was uncommon in *RET*-rearranged lung cancers in this study. Only one case treated with immunotherapy highly expressed PD-L1 (50%) and, despite this, still responded poorly to dual immune checkpoint inhibitor therapy (case 16). A recommendation regarding the use of pembrolizumab in treatment-naïve, advanced *RET*-rearranged lung cancers with high PD-L1 expression cannot be made on the basis of our findings, although its use should be approached with caution. In *MET* exon 14–altered lung cancers, TMB is similarly low; although a higher proportion of tumors have high PD-L1 expression, this was not associated with increased benefit with single-agent immunotherapy.^17^

Finally, no patients in this series received the combination chemotherapy and immunotherapy that is currently approved for the treatment of *EGFR* and *ALK* wild-type advanced NSCLC. Given the benefit of pemetrexed-containing regimens in *RET*-rearranged lung cancers, a combination of a platinum agent, pemetrexed, and pembrolizumab is reasonable to consider. Prospective trials of immunotherapy combinations should incorporate comprehensive molecular profiling to establish prospective data in driver-positive subpopulations of patients.

In summary, most *RET*-rearranged lung cancers have low PD-L1 expression and low TMB, and response to immunotherapy was not observed in this series. Although this study is limited by a small sample size, given the rarity of *RET*-rearranged NSCLCs, these findings remain meaningful and support evolving literature showing that outcomes with immune checkpoint inhibition are poor in select oncogene-addicted NSCLCs. Other systemic therapy strategies should instead be considered in patients with advanced *RET*-rearranged lung cancers before administering immunotherapy alone.

## Figures and Tables

**Table A1.**
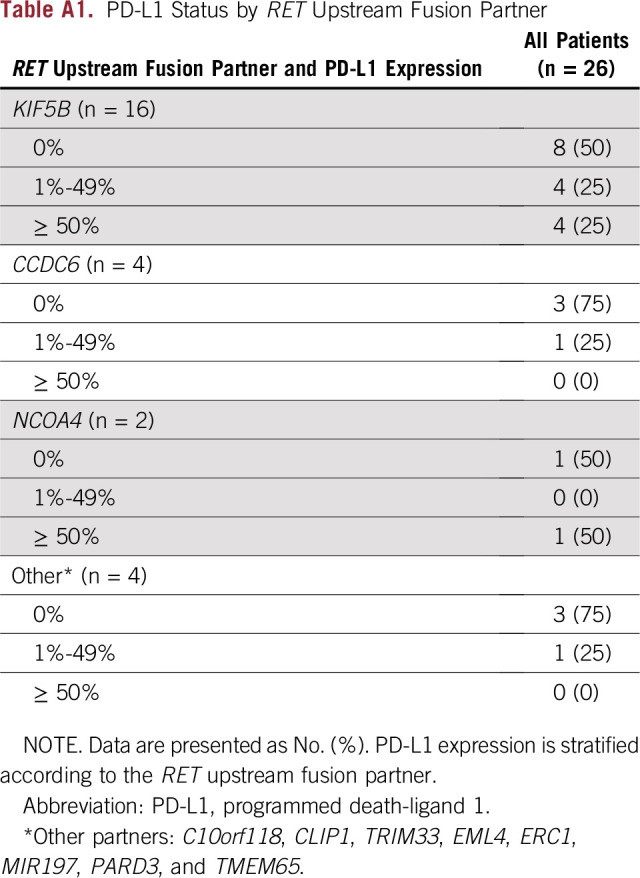
PD-L1 Status by *RET* Upstream Fusion Partner

## References

[1] Stransky N Cerami E Schalm S et al The landscape of kinase fusions in cancer Nat Commun 5 4846 2014 25204415 10.1038/ncomms5846 PMC4175590

[2] Drilon A Hu ZI Lai GGY et al Targeting RET-driven cancers: Lessons from evolving preclinical and clinical landscapes Nat Rev Clin Oncol 15 151 167 2018 29134959 10.1038/nrclinonc.2017.175 PMC7938338

[3] Drilon A Bergagnini I Delasos L et al Clinical outcomes with pemetrexed-based systemic therapies in RET-rearranged lung cancers Ann Oncol 27 1286 1291 2016 27056998 10.1093/annonc/mdw163 PMC4922319

[4] Oxnard GR Subbiah V Park K et al Clinical activity of LOXO-292, a highly selective RET inhibitor, in patients with RET fusion+ non-small cell lung cancer: An update from ASCO 2018 IASLC 19th World Conference on Lung Cancer Toronto, Canada September 23-26, 2018

[5] Subbiah V Taylor M Lin J et al Abstract CT043: Highly potent and selective RET inhibitor, BLU-667, achieves proof of concept in a phase I study of advanced, *RET*-altered solid tumors http://cancerres.aacrjournals.org/content/78/13_Supplement/CT043

[6] Drilon A Rekhtman N Arcila M et al Cabozantinib in patients with advanced RET-rearranged non-small-cell lung cancer: An open-label, single-centre, phase 2, single-arm trial Lancet Oncol 17 1653 1660 2016 27825636 10.1016/S1470-2045(16)30562-9 PMC5143197

[7] Lee SH Lee JK Ahn MJ et al Vandetanib in pretreated patients with advanced non-small cell lung cancer-harboring RET rearrangement: A phase II clinical trial Ann Oncol 28 292 297 2017 27803005 10.1093/annonc/mdw559

[8] Gandhi L Rodríguez-Abreu D Gadgeel S et al Pembrolizumab plus chemotherapy in metastatic non-small-cell lung cancer N Engl J Med 378 2078 2092 2018 29658856 10.1056/NEJMoa1801005

[9] Reck M Rodríguez-Abreu D Robinson AG et al Pembrolizumab versus chemotherapy for PD-L1-positive non-small-cell lung cancer N Engl J Med 375 1823 1833 2016 27718847 10.1056/NEJMoa1606774

[10] Hellmann MD Ciuleanu TE Pluzanski A et al Nivolumab plus ipilimumab in lung cancer with a high tumor mutational burden N Engl J Med 378 2093 2104 2018 29658845 10.1056/NEJMoa1801946 PMC7193684

[11] Cheng DT Mitchell TN Zehir A et al Memorial Sloan Kettering-Integrated Mutation Profiling of Actionable Cancer Targets (MSK-IMPACT): A hybridization capture-based next-generation sequencing clinical assay for solid tumor molecular oncology J Mol Diagn 17 251 264 2015 25801821 10.1016/j.jmoldx.2014.12.006 PMC5808190

[12] Zheng Z Liebers M Zhelyazkova B et al Anchored multiplex PCR for targeted next-generation sequencing Nat Med 20 1479 1484 2014 25384085 10.1038/nm.3729

[13] Gaule P Smithy JW Toki M et al A quantitative comparison of antibodies to programmed cell death 1 ligand 1 JAMA Oncolepub ahead of print on August 18, 2016 10.1001/jamaoncol.2016.3015 PMC5491359 27541827

[14] Rizvi H Sanchez-Vega F La K et al Molecular determinants of response to anti-programmed cell death (PD)-1 and anti-programmed death-ligand 1 (PD-L1) blockade in patients with non-small-cell lung cancer profiled with targeted next-generation sequencing J Clin Oncol 36 633 641 2018 29337640 10.1200/JCO.2017.75.3384 PMC6075848

[15] Hellmann MD Nathanson T Rizvi H et al Genomic features of response to combination immunotherapy in patients with advanced non-small-cell lung cancer Cancer Cell 33 843 852.e4 2018 29657128 10.1016/j.ccell.2018.03.018 PMC5953836

[16] Offin M Rizvi H Tenet M et al Tumor mutation burden and efficacy of EGFR-tyrosine kinase inhibitors in patients with EGFR-mutant lung cancers Clin Cancer Res 25 1063 1069 2019 30045933 10.1158/1078-0432.CCR-18-1102 PMC6347551

[17] Sabari JK Leonardi GC Shu CA et al PD-L1 expression, tumor mutational burden, and response to immunotherapy in patients with MET exon 14 altered lung cancers Ann Oncol 29 2085 2091 2018 30165371 10.1093/annonc/mdy334 PMC6225900

[18] Mazieres J Drilon AE Mhanna L et al Efficacy of immune-checkpoint inhibitors (ICI) in non-small cell lung cancer (NSCLC) patients harboring activating molecular alterations (ImmunoTarget) J Clin Oncol 36 2018 (suppl; abstr 9010)

[19] Subbiah V Gainor JF Rahal R et al Precision targeted therapy with BLU-667 for *RET*-driven cancers Cancer Discov 8 836 849 2018 29657135 10.1158/2159-8290.CD-18-0338

